# Automated Detection of Lupus White Matter Lesions in MRI

**DOI:** 10.3389/fninf.2016.00033

**Published:** 2016-08-12

**Authors:** Eloy Roura, Nicolae Sarbu, Arnau Oliver, Sergi Valverde, Sandra González-Villà, Ricard Cervera, Núria Bargalló, Xavier Lladó

**Affiliations:** ^1^Department of Computer Architecture and Technology, University of GironaGirona, Spain; ^2^Centre de Diagnòstic per la Imatge, Hospital ClínicBarcelona, Spain; ^3^Department of Autoimmune Diseases, Hospital Clínic-Institut d'Investigació Biomèdica August Pi i SunyerBarcelona, Spain; ^4^Magnetic Resonance Imaging Core Facility, Institut d'Investigació Biomèdica August Pi i SunyerBarcelona, Spain

**Keywords:** magnetic resonance images, lupus disease, image analysis, automatic lesion detection and segmentation

## Abstract

Brain magnetic resonance imaging provides detailed information which can be used to detect and segment white matter lesions (WML). In this work we propose an approach to automatically segment WML in Lupus patients by using T1w and fluid-attenuated inversion recovery (FLAIR) images. Lupus WML appear as small focal abnormal tissue observed as hyperintensities in the FLAIR images. The quantification of these WML is a key factor for the stratification of lupus patients and therefore both lesion detection and segmentation play an important role. In our approach, the T1w image is first used to classify the three main tissues of the brain, white matter (WM), gray matter (GM), and cerebrospinal fluid (CSF), while the FLAIR image is then used to detect focal WML as outliers of its GM intensity distribution. A set of post-processing steps based on lesion size, tissue neighborhood, and location are used to refine the lesion candidates. The proposal is evaluated on 20 patients, presenting qualitative, and quantitative results in terms of precision and sensitivity of lesion detection [True Positive Rate (62%) and Positive Prediction Value (80%), respectively] as well as segmentation accuracy [Dice Similarity Coefficient (72%)]. Obtained results illustrate the validity of the approach to automatically detect and segment lupus lesions. Besides, our approach is publicly available as a SPM8/12 toolbox extension with a simple parameter configuration.

## 1. Introduction

Several brain diseases present abnormalities in the white matter tissue, usually denoted as white matter lesions (WML). Segmenting these WML is important to diagnose and better understand these diseases as well as monitoring its progression. However, performing this task manually is tedious and very time consuming. Hence, several works have been proposed to tackle automatically this lesion segmentation problem. For instance, various approaches have been presented in multiple sclerosis lesion segmentation (Van Leemput et al., 2001; Lladó et al., 2012a,b; Schmidt et al., 2012; Cabezas et al., 2014a,b; Guizard et al., 2015; Jain et al., 2015; Roura et al., 2015; Brosch et al., 2016), stroke (Mitsias et al., 2002), vascular dementia (Mohamed et al., 2001; Yamashita et al., 2008), and other diseases (Kruggel et al., 2008; Schwarz et al., 2009). Instead, few attempts have been done on semiautomatic or automatic segmentation of Lupus lesions (Appenzeller et al., 2008a,b; Petri et al., 2008; Scully et al., 2010), which have the particularity of being very small and focal WML, and they are few and isolated.

Magnetic resonance imaging (MRI) is the gold standard technique for studying the brain in lupus (Sarbu et al., 2015c). The neuroimaging findings are classified as small or large vessel disease, and inflammatory-type lesions (Sarbu et al., 2015b). Small vessel disease is represented by white-matter hyperintensities/lesions, recent small subcortical infarcts, lacunes, microbleeds, and brain atrophy (Wardlaw et al., 2013). WML are the most common findings of small vessel disease seen in lupus, and represent small T2-hyperintensities following the distribution of the white matter (periventricular, deep, subcortical), and including also the white matter at the basal ganglia, and cerebellum (Sarbu et al., 2015b,c). During the last years, WML have been shown to function as an independent predictor for the neurolupus activity and injury, and quantitative methods are increasingly proposed for the quantification and follow-up of the WML in neurolupus. As stated in Sarbu et al. (2015b), the stratification by the number of lesions is also important for the diagnosis of neuropsychiatric lupus. In their work, the authors introduced an stratification in three different groups: (1) low lesion burden (< 5 lesions); (2) medium lesion burden (between 5 – 25 lesions); and (3) high lesion burden (> 25 lesions).

To deal with the lupus WML segmentation problem, previous approaches, such as the automated one of Scully et al. (2010), have used a supervised strategy. In their work, local morphometric features extracted from multiple sequences, including T1w, T2w, and fluid-attenuated inversion recovery (FLAIR) images, were used to train a supervised classifier that takes advantage of a different subset of the features to segment lesion voxels. With a different viewpoint, in our work we present an unsupervised approach to automatically segment WML in Lupus patients by using only T1w and FLAIR images. This work can be seen as an extension of the tool recently presented by Roura et al. (2015), in which the focus was the segmentation of multiple sclerosis lesions. The whole pipeline can be considered as a two step process: pre-processing and WML segmentation. The first step is focused on the image enhancement by performing different intensity corrections on the brain and co-aligning all the image modalities. The second one, performs the lesion segmentation by detecting outliers to the normal apparent gray matter brain tissue on the FLAIR image as was previously done by Souplet et al. (2008) and Roura et al. (2015). Given the specific properties of the Lupus WML, we introduce a set of post-processing steps to reduce possible false positive (FP) detections which are based on lesion size, lesion tissue neighborhood, and lesion location. The last one aims to eliminate the FP detections usually found in the posterior fossa due to frequent scanner artifacts, yet this is an uncommon location for WML in neurolupus (up to 7% of patients vs. 40–60% in frontal lobes) (Sarbu et al., 2015b). We introduced this constraint in the segmentation by using an in-house atlas created with the unbiased template creation algorithm proposed by Fonov et al. (2011), which was then segmented into 12 brain structures including the posterior fossa using the Computational Morphometry Toolkit software^1^.

The evaluation of the Lupus WML segmentation has been done on a dataset of 20 patients comparing quantitatively the results obtained by our tool with the ones performed manually by an expert neuroradiologist. This ground truth (GT) has been used to compute quantitative measures in terms of detection, such as True Positive Rate (TPR) and Positive Prediction Value (PPV), and in terms of segmentation accuracy by using the Dice Similarity Coefficient (DSC). Both detection and segmentation results show the ability of the approach to automatically detect and segment focal WML in Lupus patients. The code of our approach is publicly available as a Statistical Parametric Mapping (SPM8/12) toolbox extension with a simple parameter configuration^2^.

## 2. Materials and methods

### 2.1. Data

This study included 20 Lupus patients. The brain MRIs were performed between 2014 and 2015 at Hospital Clínic, University of Barcelona, Spain, the main national referral institution for lupus. All scans were performed at three Tesla Siemens MAGNETOM TIM Trio scanner, using a 32-channel head coil, with the same protocol including 3D T1 and 3D FLAIR, with a voxel size = 1 × 1 × 1*mm*^3^. The lesions were semiautomatically annotated on FLAIR images by an expert neuroradiologist. They present a lesion volume mean and range (min-max) per patient of 0.217 [11−1459] *mm*^3^. This study was carried out in accordance with the ethical recommendations of the Hospital Clínic committee (IDIBAPS, Barcelona), with written informed consent from all subjects.

### 2.2. MRI pre-processing

**Figure 1:**
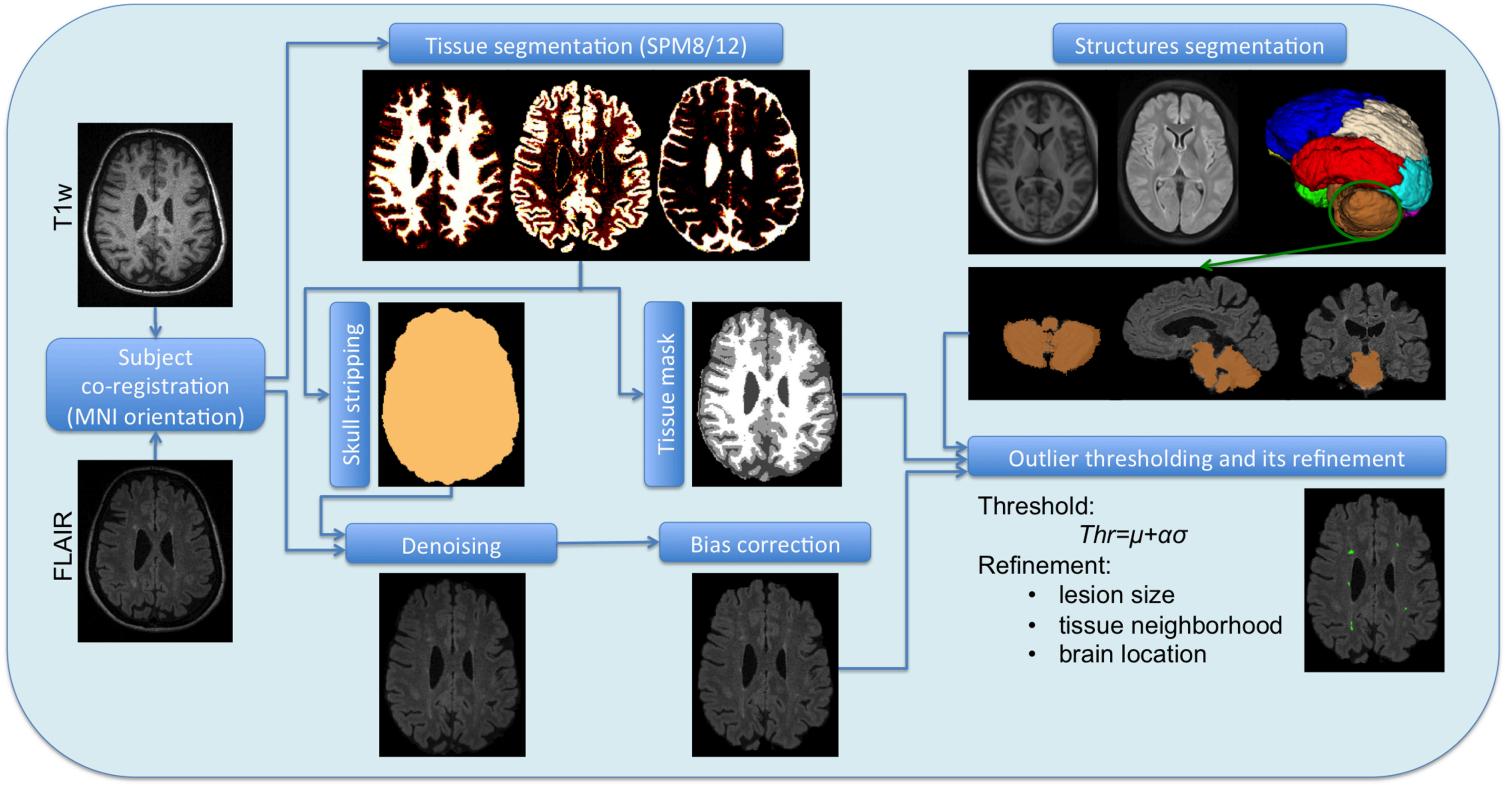
**Scheme of the full pipeline**. T1w and FLAIR images are the original subject images, which require a co-registration. Once co-registered they undergone a separate pre-processing, T1w by SPM8/12 to obtain the tissue segmentation and the brain mask, while FLAIR is denoised and unbiased by separate methods. The atlas and its structures also belong to the SPM space, so they can be brought to the subject space as the tissue segmentation and the brain mask. The corrected FLAIR image, the tissue segmentation and the posterior fossa mask are the inputs of the WML segmentation tool.

To deal with the Lupus WML segmentation, several pre-processing steps (see Figure 1) are required to optimize the overall performance, as seen in previous works (Schmidt et al., 2012; Cabezas et al., 2014a,b; Valverde et al., 2014; Guizard et al., 2015; Roura et al., 2015). Since, our aim is to provide a publicly available Lupus segmentation tool as an extension of the SPM8/12 all the required steps are performed within the Matlab environment.

The first step of the pre-processing consists in the intra-subject registration. For this, we follow a similar procedure as the one used in Roura et al. (2015). In this case, we register FLAIR to the T1w image, where the target space used [corresponding to the Montreal Neurological Institute (MNI) (Mazziotta et al., 1995)] as well as the co-registration software are provided by the SPM toolbox.

One of the most common pre-processing step is the skull stripping process (Shattuck et al., 2001; Smith, 2002; Park and Lee, 2009; Roura et al., 2014), which we incorporate into our pipeline using the SPM tissue segmentation algorithm (Ashburner and Friston, 2005), avoiding therefore the use of external libraries such as BET (Smith, 2002) or BSE (Shattuck et al., 2001). Given that this process provides the probability map of the three main brain tissues [white matter (WM), gray matter (GM), and cerebrospinal fluid (CSF)], we created a maximum probability map to determine the three tissue masks and then thresholded the result at 0.5 to determine directly the brain mask. A similar procedure was also applied in previous segmentation works (Boesen et al., 2004; Roura et al., 2014). Notice that a threshold of 0.5 could lead to some holes in the corresponding masks due to the signal changes induced by the presence of WML. While partial volume effects will have only two tissue classes competing (WM/GM or GM/CSF), in the presence of lesions, areas such as periventricular, are prone to misclassifications among the three main tissue classes. In this case, a morphological operation should be applied in order to fill the inside holes of the brain mask. However, this is very unlikely and we did not experience this when using the SPM12 platform. This process is performed on the T1w image, although the brain mask is then applied on the FLAIR image where the rest of the pre-processing steps are carried out, since intensity corrections of the T1w image are handled by the SPM tissue segmentation process itself.

It is well-known that MRI images obtained directly from the scanner present noise and undesired artifacts (movement, high signal value, blood, flow artifacts, etc) (Lemieux et al., 1998). These abnormalities such as inhomogeneities in the magnetic field (Sha and Sutton, 2001) must be attenuated by applying post-scanning processes. We first apply the anisotropic diffusion filter of Perona and Malik (1990) in order to enhance the image by smoothing its histogram with the 3D Matlab implementation^3^ of this algorithm. Given the reduced size of the lesions, we have carefully run this method over all the patients with a restrictive parameter configuration (1 iteration, *K* = 50, and high contrast edges), reducing the iterations and gradient modulus, and focusing on contrast instead of region size.

To correct the bias field we used the Matlab method proposed by Larsen et al. (2014), which is based on an expectation maximization model (EM) that relies on the same generative models and bias field estimation computations of the well-known non-parametric, non-uniform intensity normalization (N3) method (Sled et al., 1998). This approach requires to mask out the low intensity voxels, thus the brain mask obtained from T1w image is used when correcting the FLAIR image.

### 2.3. Lupus lesion segmentation

**Figure 2:**
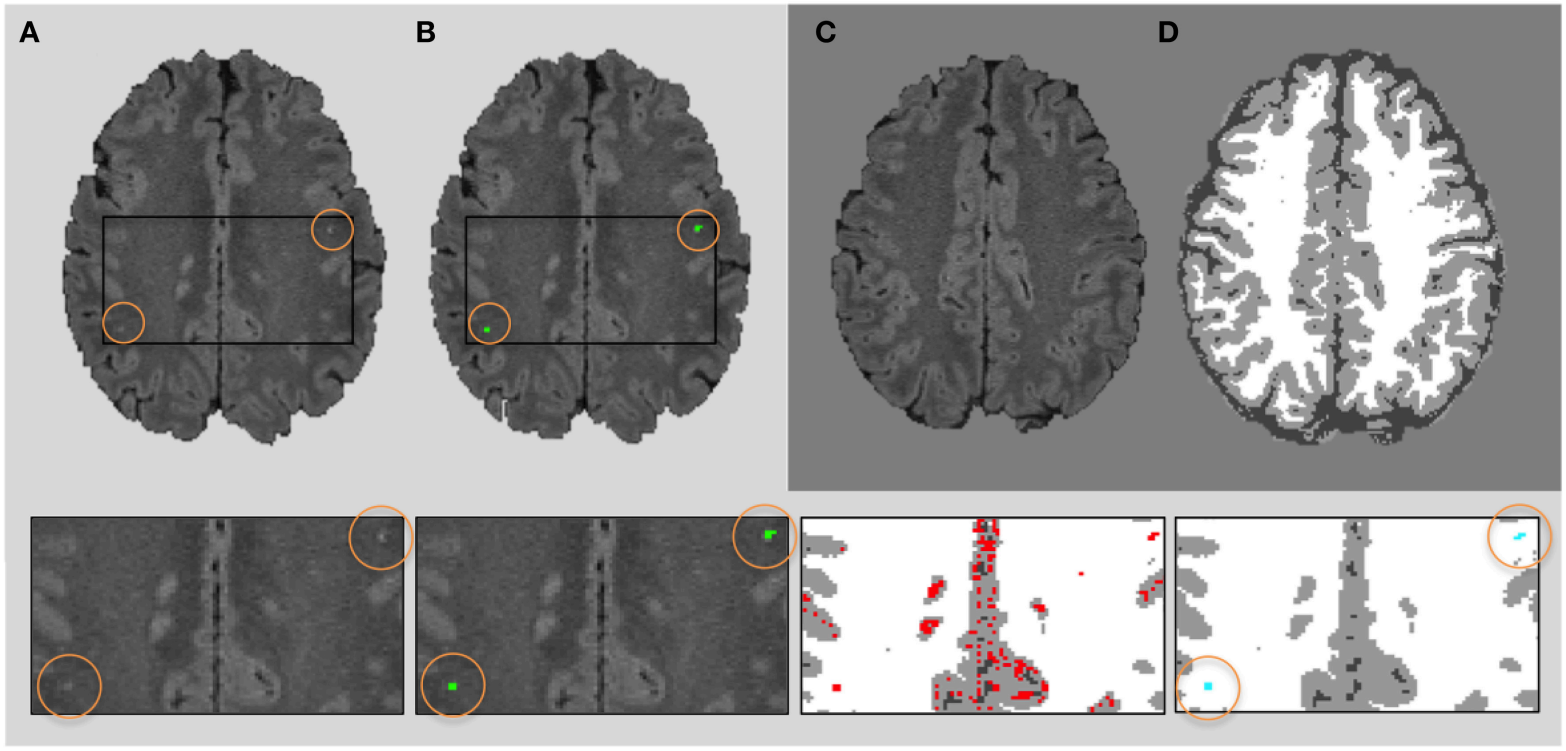
**Neighboring rule**. FLAIR 2D axial slice **(A)** showing 2 lesions (in green) of 4 and 5 voxels, respectively **(B)**, both completely surrounded by white matter. Original image and tissue segmentation result of two slices forward are shown in **(C,D)**. The bottom row shows four zooms of the original image, ground truth (green), candidates regions (red), and final lesion segmentation (blue).

Lupus lesions, similarly to other WML such as multiple sclerosis lesions, are characterized by being hyperintense regions in the FLAIR images. Due to the fact that the GM is the highest intensity tissue in this image modality, we used its histogram distribution to identify the hyperintense outliers. In order to obtain the GM distribution, we used the same SPM tissue segmentation (Ashburner and Friston, 2005) applied in the skull stripping process. At this point the lesion detection can be performed as a thresholding process, commonly computed by μ+ασ, where the standard deviation (σ) is determined using the full width at half maximum (FWHM) of the main peak (μ). We can then adjust the number of detected candidate lesions via the α parameter, observing a good trade off when setting this parameter to 2.5, assuming more than 98% of the histogram belonging to GM. Afterwards, we apply a set of post-processing steps to remove FP lesions that remained after thresholding the FLAIR image: (1) Lesion size: we constraint the minimum size of the lupus lesion to be 3 *mm*^3^. Therefore, we eliminate hyperintense voxels or a group of voxels smaller than this size. (2) Lesion tissue neighborhood (λ): because the lupus lesions should appear in the WM, the surrounding voxels must strictly belong to WM. Therefore, we introduce a parameter to limit the proportion of the WM over GM and CSF in the lesion neighborhood. We will see in Section 3 that the best trade off was obtained when using λ = 0.7. Looking at Figure 2, one can see how the neighbors of the two higher hyperintense regions marked in green in Figure 2B, all belong to WM in the tissue segmentation, while other candidate regions seen in the centre (marked in red) are not considered lesions because the neighbors voxels belong to GM. This neighborhood operation is applied in 3D. Figures 2C,D shows the original image and the tissue segmentation result of two slices forward, where the candidates marked in red are attached to GM and therefore eliminated with the neighborhood constraint. (3) Lesion location: since Lupus lesions are rarely present in the posterior fossa (Sarbu et al., 2015b,c), and this particular area is highly prone to present hyperintense artifacts, we have decided to exclude this region when looking for possible lesion candidates. This is done automatically by registering an atlas with the corresponding structure segmentation to the T1w image. In particular, we use an in-house 3T template created over healthy subjects using the unbiased template creation approach proposed by Fonov et al. (2011). This procedure, as stated by the authors, converges after 20 iterations, meaning that 20 non-rigid registrations must be performed for each subject of the population. The nonlinear registration process relies on the Automatic Nonlinear Image Matching and Anatomical Labeling (ANIMAL) of Collins et al. (1995). In order to obtain the structure segmentation of the healthy template, we have re-arranged the 83 labels of the T1w atlas from Hammers et al. (2003) into 12 regions^4^. Subsequently, our template was segmented into these 12 regions using the Computational Morphometry Toolkit (CMTK)^5^. To register the in-house template T1w image to each patient we used the SPM registration module, similarly to the intra-subject registration process. Finally, using the deformation field obtained by the non-rigid registration, we are able to bring the structure corresponding to the posterior fossa to each of the patient's space and therefore remove FP in this area caused by artifacts. A summary of the full pipeline is illustrated in Figure 1.

## 3. Results

We have quantitatively analyzed the obtained results evaluating three different measures, TPR and PPV for lesion detection, sensitivity and precision, respectively, and DSC in terms of segmentation accuracy. The evaluation of our approach has been carried out through a two-fold cross-validation method where 10 random samples have been chosen for the first set while the rest have been used in the second set. For each training step we have exhaustively assessed the parameter configuration by computing a trade-off between DSC, TPR, and PPV using the traditional F-score measure (Zhang et al., 2015), which gives the harmonic mean of the three measures abovementioned as follows:

(1) $$\begin{array}{l} F\text{-}score=3\times \frac{DSC\times TPR\times PPV}{DSC+TPR+PPV} \end{array}$$

**Figure 3:**
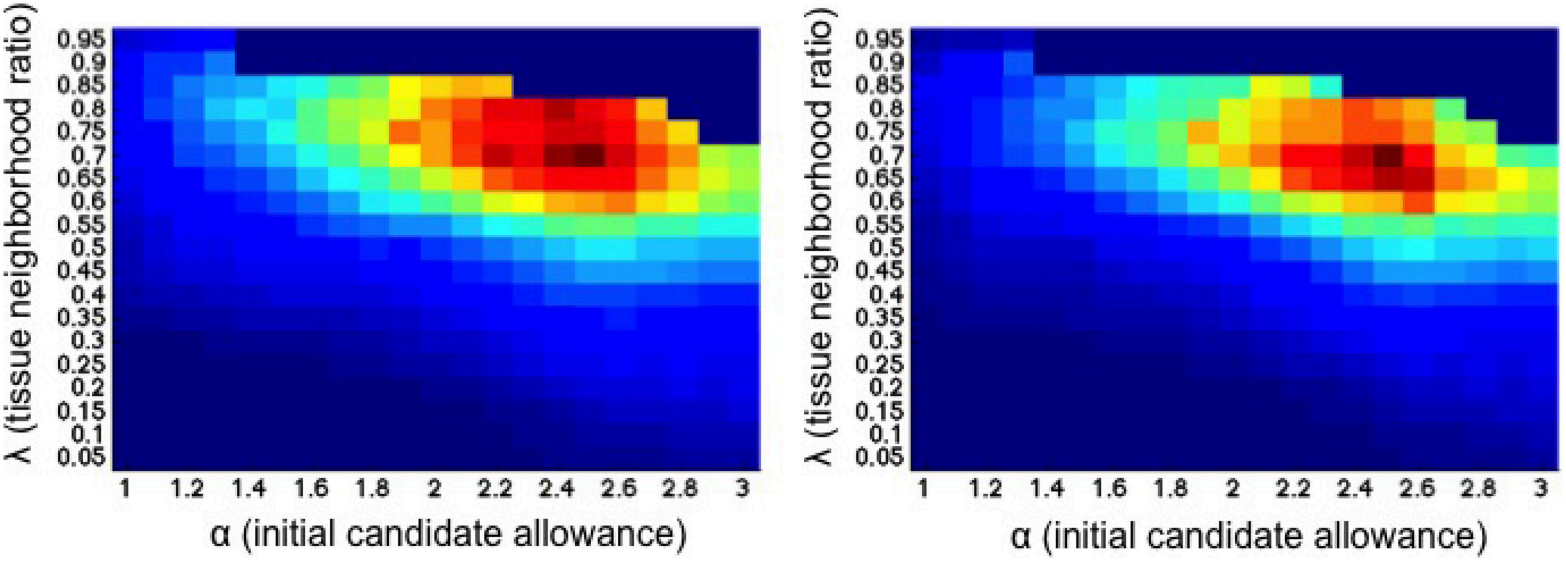
**Joint evaluation of both α and λ parameters (x and y axes, respectively) for each training set within the two-fold cross-validation**. Each position in the map represents the mean F-score for a specific tissue neighborhood ratio λ and the initial candidate lesions adjustment α. Reddish colors show higher mean F-score values.

A logical range of parameters has been tested for both α and λ (x and y axes, respectively in Figure 3), on each training set of the 10 patients randomly chosen within the two-fold cross-validation. The best configuration was α = 2.5 and λ = 0.70 for both sets, therefore these parameters were used in all the 20 patients of the dataset obtaining the results presented here.

**Figure 4:**
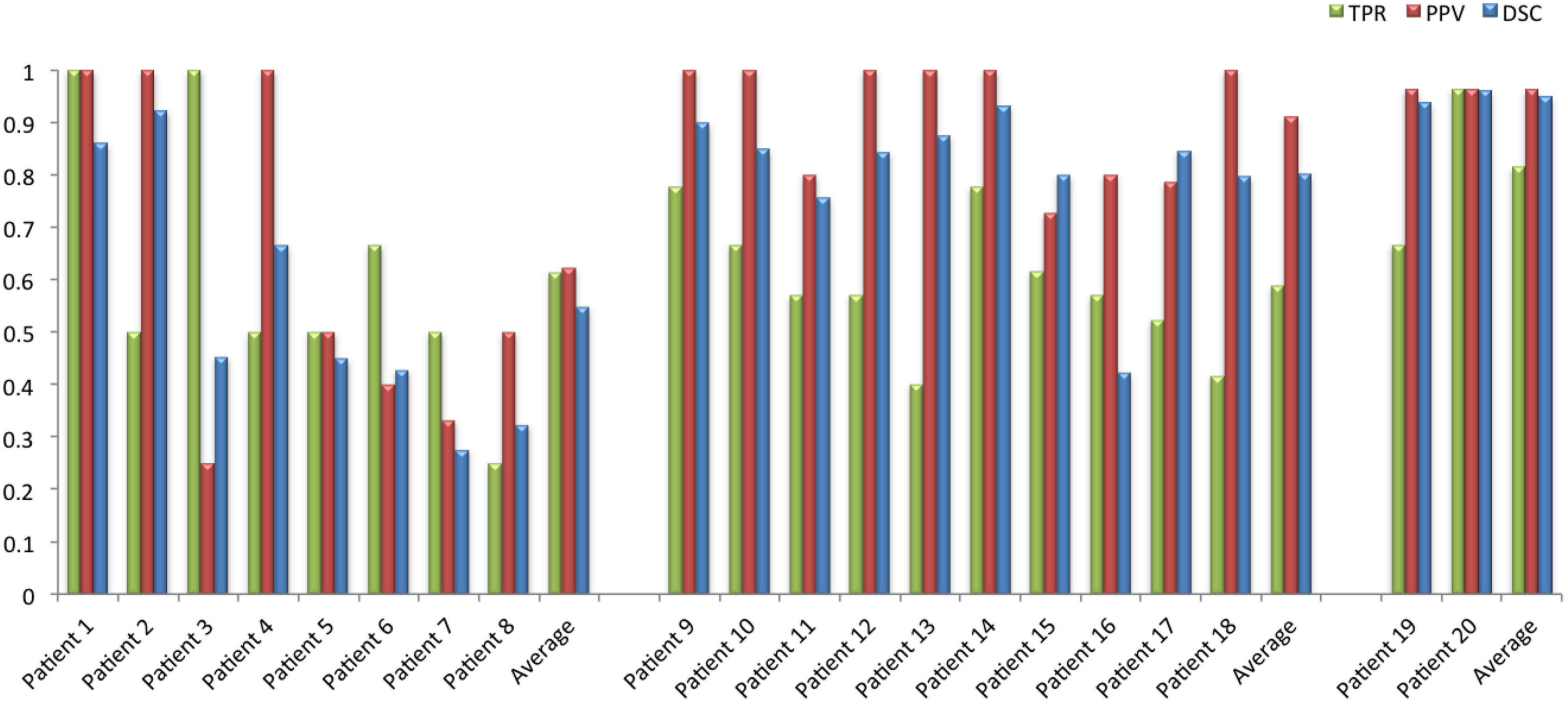
**Bar plots of each patient representing the DSC, TPR, and PPV values**. The population is stratified in three groups depending on the GT number of lesions, from left to right: < 5; [5−25]; >25 lesions per patient.

Figure 4 shows the obtained results per patient. As done in Sarbu et al. (2015b), we have stratified the population according to three different groups depending on the number of GT lesions per patient: (1) low lesion burden (< 5 lesions); (2) medium lesion burden (between 5 – 25 lesions); (3) high lesion burden (>25 lesions). Notice that this stratification by number of lesions may help on the diagnosis of neuropsychiatric lupus. Regarding the obtained results, both group and total averages of all the measures are over 50%, specially highlighting the group with more than 25 lesions, where we obtain a *TPR* = 0.81±0.14, a *PPV* = 0.96, and a *DSC* = 0.95±0.1. Notice, however, that this particular group only contains two samples and therefore we cannot extract significant conclusions. When considering the whole dataset, these values are: *TPR* = 0.62±0.19, *PPV* = 0.80±0.25, and *DSC* = 0.72±0.22; this decrease is due to the lower performance obtained in the first group of the stratification, where a small error represents a big percentage in the total measure. These cases with <5 lesions have also a small lesion volume which as stated in previous studies (Roura et al., 2015) worsen the performance of the automated segmentation methods.

**Figure 5:**
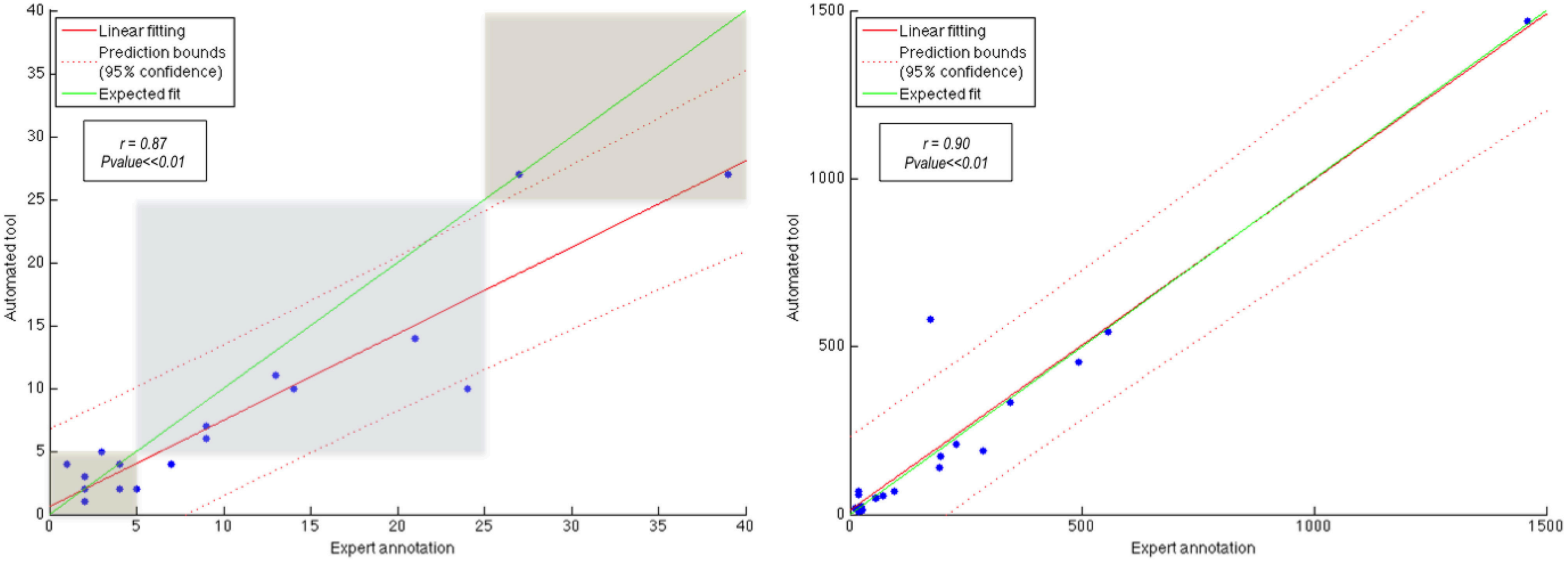
**Correlation with number of lesions (stratified by the three groups) on the left and lesion volume, in terms of voxels, on the right**.

**Figure 6:**
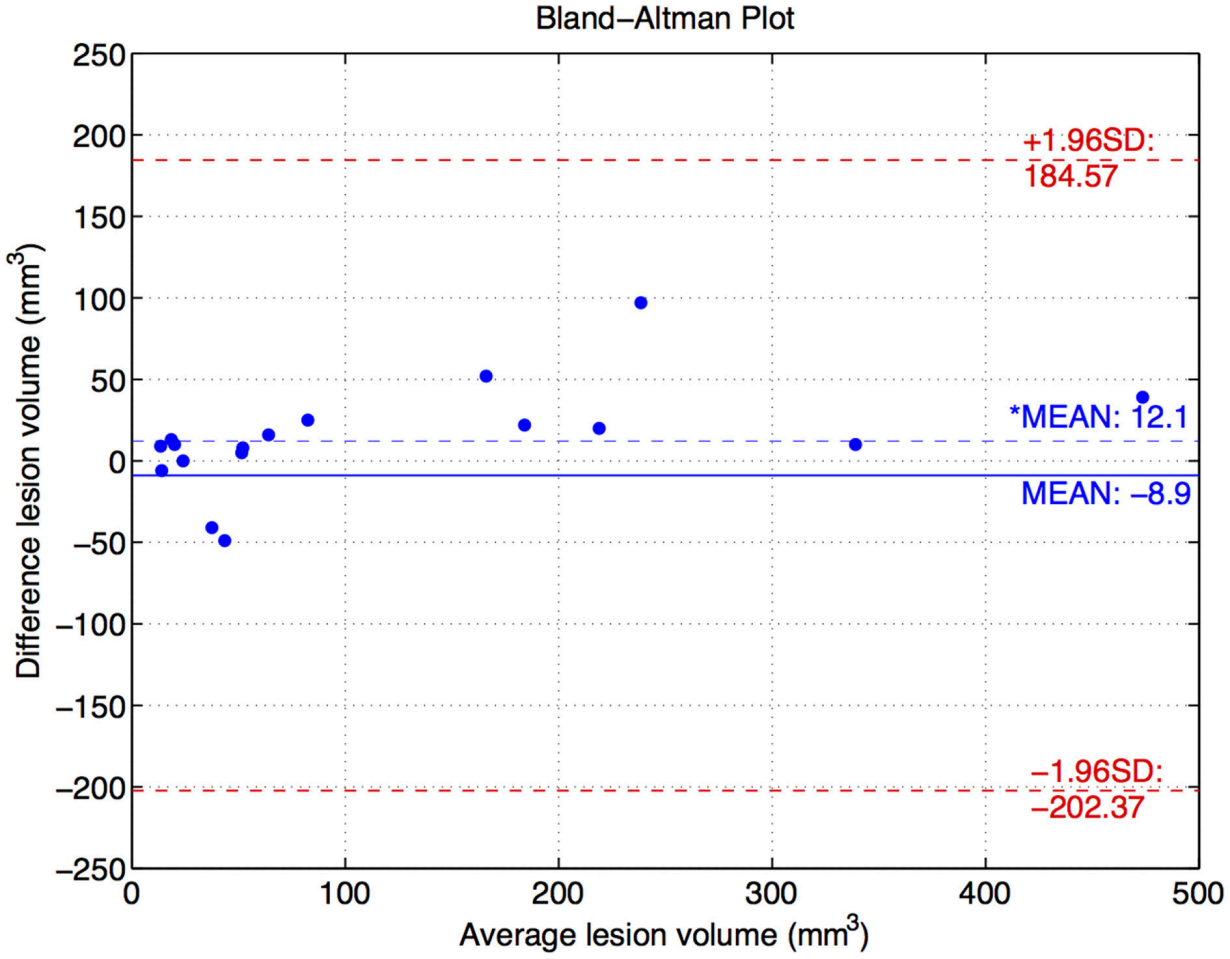
**Bland-Altman plot of the lesion volume for those cases below 500 *mm*^3^ of average volume**. There is also one outlier not represented with 400 *mm*^3^ of difference volume and 370 *mm*^3^ of average volume. Besides, two scatter points over 500 *mm*^3^ of average volume have also been removed, although the difference volume was 10 *mm*^3^ for both. The* represents the mean difference volume when removing the outlier from the analysis.

To better understand the results, we also show two correlation plots, one with the number of lesions and one with the lesion volume (see Figure 5). In order to evaluate these correlations we first tested the normality of the data using the Lilliefors test based on the Kolmogorov-Smirnov normality test (Lilliefors, 1967). Given that the distributions did not follow a normal distribution we applied the Spearman's linear correlation coefficient. We have fitted a linear polynomial curve and showing also the expected fit, which is basically the ideal correlation. Looking at the number of lesions correlation, the model lies under the expected fit meaning that the approach underestimates the number of lesions. However, we obtained a very high linear correlation coefficient (*r* = 0.87) with a *p* ≪ 0.01, meaning that the coefficient is significantly different from zero, and therefore the null hypothesis of no correlation is not satisfied. The whole dataset can be linearly explained because all the samples follow the same trend, except one outlier which also has a correct stratification. Besides, one can see how the stratification results fit for most of the patients with the expected groups, except for two cases which are close to the group limit. Regarding the lesion volume correlation, the model fitting shows also a very good correlation, with only one sample out of the confidence level. The model coincides almost perfectly with the expected fit and Spearman's coefficient is also high with *r* = 0.90 with a *p* ≪ 0.01. Notice that this high fitting illustrates that the FP and FN are not significant compared to the TP in terms of total affected tissue volume, where the *FNR* (0.19±0.18) in terms of volume was relatively low with respect to the *TPR* (0.81±0.18), i.e., the lesions missed by the approach presented a very small volume and therefore the overall correlation per patient was high. To better understand the behavior on the low lesion volume per patient, we also show the Bland-Altman plot (Figure 6), where one can better perceive the good correlation of the scatter points skewed to the left in the first volume correlation plot. For a better visualization, we have also removed the two scatter points over 500 *mm*^3^ and also the outlier, which in fact shows a difference volume of around 400 *mm*^3^ while the other two scatter points lies close to the mean volume difference (10 *mm*^3^). Even though the mean difference volume was −8.9 *mm*^3^, indicating an overall oversegmentation trend of our tool, this is mainly due to the outlier case in which we have a very big overestimation of the total volume. In fact, when removing this outlier from the analysis the mean difference volume value changes to be 12.1 *mm*^3^ (see doted blue line in Figure 6), which indicates a general trend of our tool to undersegment the lesion volume. This reflects also the trend with the volume correlation plot shown in Figure 5. Furthermore, we have also analyzed the behavior individually for the 3 different groups depending on the number of lesions per patient: (1) < 5 lesions: mean volume difference of −7 *mm*^3^ (oversegmentation) with a mean number of lesions per patient of 2.5; (2) between 5 – 25 lesions (excluding the outlier): mean volume difference of 28.5 *mm*^3^ (undersegmentation) with a mean number of lesions per patient of 12.5; (3) >25 lesions: mean volume difference of 14.5 *mm*^3^ (undersegmentation) with a mean number of lesions per patient = 33. Therefore, from this analysis, we can point out that our tool tends to oversegment the cases with a very small lesion volume and number of lesions, in fact the extreme cases in which there is more confusion with possible artifacts and partial volume effects, while is more conservative (undersegmentation) with the rest of cases in which there are larger number of lesions and volume.

**Figure 7:**
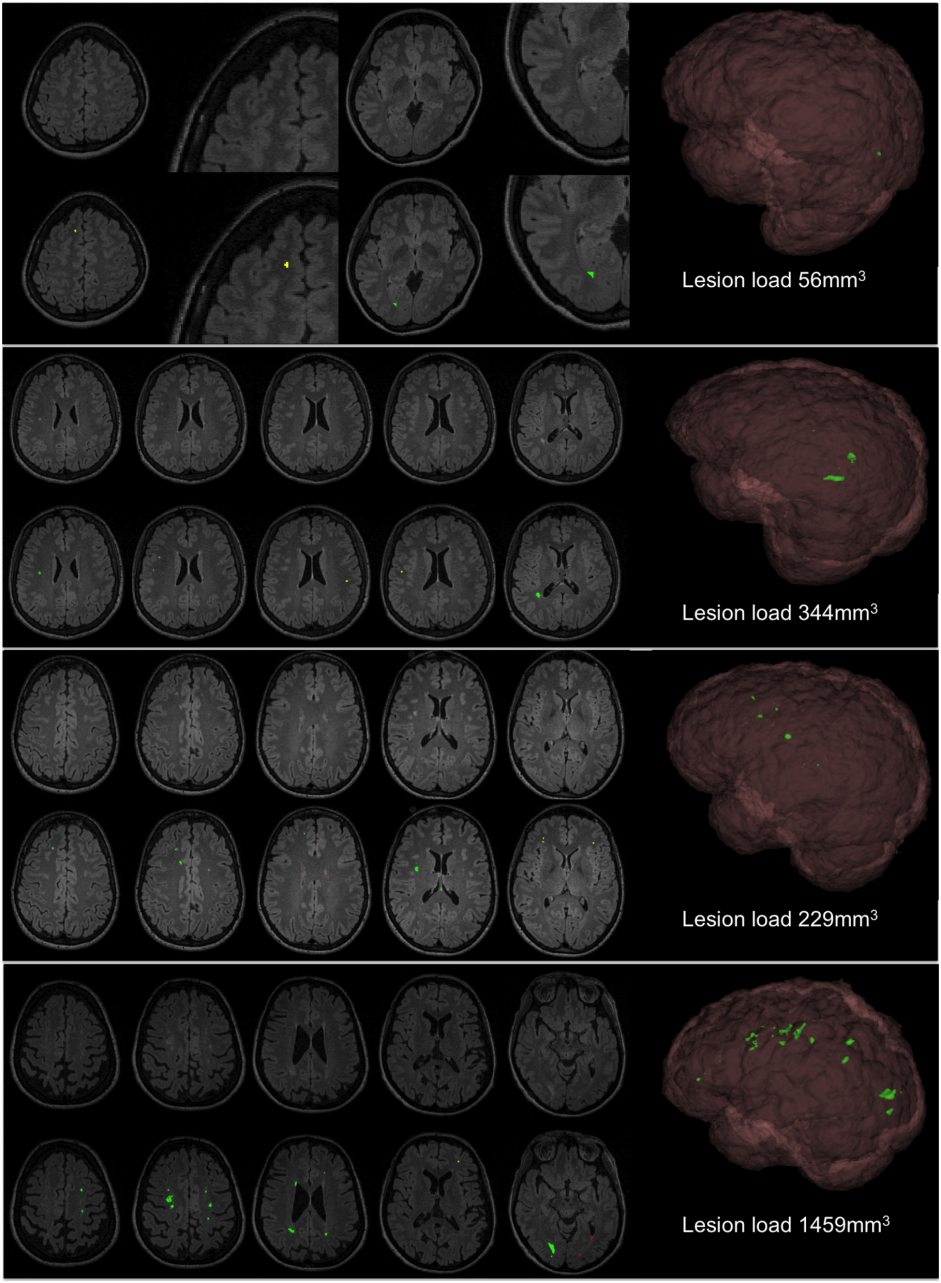
**Qualitative results of the approach**. First row of each patient shows the original FLAIR image and second row shows the automatic segmentation (green, TP; red, FP; and yellow, FN).

Some samples of qualitative results are shown in Figure 7, where we compare the results of our automated tool with the GT annotations. We have chosen different samples to illustrate the performance in patients with different lesion load. Notice that the total lesion volume is very small in all of them, but the automatic detection provides a good performance in terms of TP while having a reduced number of FP and FN. When illustrating the whole 3D volume in the figure, those FP and FN are inappreciable because they are smaller than (10 *mm*^3^). However, we show some FP and FN examples on the 2D slices for the second and third group, zooming also into these regions in the first group.

The three cases shown in Figure 7 are the most representative for the FNs found within the juxtacortical area. As explained in the methodology, contextual information, and lesion size are the restrictions to filter the potential candidates, and both have to be accomplished. Given the small size of these juxtacortical lesions, a high initial threshold that only considers the focus of the lesions may fail at any of both requirements if they are low intense with respect to the rest. Although it can be solved increasing the α parameter, this could lead to higher FP detections in other regions.

In order to compare our approach with the state-of-the-art (Scully et al., 2010), we have also analyzed the performance of our approach in terms of sensitivity and specificity for some specific points of the receiver operating characteristic (ROC) curves, given that the combination of two parameters within a ROC curve is not trivial. In the work of (Scully et al., 2010), the authors reported a sensitivity of 94.3% and a specificity of 93.9% in the hold-out set at the lowest threshold. On the other hand, they obtained 100% specificity and 2.6% sensitivity when the highest threshold shown in the ROC curve was used. At the 80% of sensitivity (the midpoint of the curve), the reported specificity was around 99.5%, and at 90% sensitivity was close to 98% specificity. In our experimental results, we have increased the specificity maintaining similar sensitivity values. In particular, we observed 100% specificity at 3% of sensitivity using our approach, while at 80% of sensitivity the specificity was 99.99%. Besides, at 90% sensitivity our specificity was still higher than 99%. Notice that a specificity of 99% might still have large FP detections, since the brain volume is much larger than lesions volume.

## 4. Discussion

WML are the most common radiological finding in neurolupus. They are non-specific findings, being frequently observed in older age groups, migraine, chronic diseases, heart diseases, diabetes, high-blood pressure, dyslipidemia, and other vascular risk factors, although they are also present in asymptomatic subjects without known diseases (Sarbu et al., 2015c). However, WML are found in 40–60% of neurolupus patients, even at the onset of the disease, and many previous reports showed a higher frequency of WML in neurolupus when compared with lupus without neurolupus and general population (Castellino et al., 2008; Wardlaw et al., 2013; Sarbu et al., 2015b,c).

The pathogenesis of WM hyperintensity is attributed to chronic small vessel disease, which is supported by a study with radiologic-pathologic correlation in patients with neurolupus (Sibbitt et al., 2010). The underlying mechanisms for small vessel disease in neurolupus are not well understood, although multiple factors are incriminated, including accelerated atherosclerosis, direct immune mediated alterations, microembolisms, intimal hyperplasia, erythrocytes extravasation, fibrin thrombi, and coagulopathy (Joseph and Scolding, 2009; Sarbu et al., 2015a,c).

In neurolupus, WML involve preferentially the frontal and parietal regions, different from primary autoimmune demyelinating diseases such as multiple sclerosis. WML were repeatedly correlated with lupus duration, cognitive dysfunction, cerebrovascular syndrome, seizures, antiphospholipid antibody and low complement (C3, C4, CH50) levels (Ainiala et al., 2005; Appenzeller et al., 2008b; Toledano et al., 2013). A quantitative WML analysis in lupus patients demonstrated that age, duration of neuropsychiatric manifestations and total corticosteroid dosage were independent predictors for WML (Appenzeller et al., 2008b). Importantly, there was demonstrated a positive association between the lesion burden and the score of lupus activity (Systemic Lupus Erythematosus Disease Activity Index-SLEDAI) and injury (Systemic Lupus International Collaborating Clinic-SLICC). This means that WML are an independent predictor for lupus activity and injury, and suggests that the quantification of WML (either by number or, maybe better, by volumetric methods) and their follow-up, could be used for monitoring the disease progression and response to therapy (Appenzeller et al., 2008a,b; Sarbu et al., 2015c).

We have proposed in this work an automated tool which presents a good correlation in both number of lesions and lesion volume, as seen in Figure 5. Even though the obtained results tend to underestimate the lesion detections, the number of lesions detected have shown a good correlation with the stratified population into three groups. Notice that the FN rate has a weak influence on the final lesion volume, since Lupus WML are small focal lesions, characteristic of this particular disease. Besides, in order to reduce this FP detection rate introduced by the artifacts of the scanner, we have set a high lesion neighborhood restriction to belong to WM.

The parameter configuration has been set up with an exhaustive analysis over both α and lesion tissue neighborhood parameters, testing values from 1 to 3 each 0.1 and from 0 to 1 each 0.05, respectively. The analysis showed that the optimal configuration was with the α of 2.5 and tissue neighborhood ratio of 0.7. We want to remark that this will be the default configuration of the tool, however, other configurations provide very similar results. After performing this evaluation, we observed how the approach was fairly steady for a wide range of parameters. Moreover, as shown in the work of Roura et al. (2015), the use of the same parameters in a similar intensity thresholding strategy, provided also promising results when tested with more than 100 multiple sclerosis patient scans acquired from different machines (3T and 1.5T).

Even though this study has been evaluated with a dataset of 20 patients, we observed promising results in both lesion detection and segmentation, highly comparable to the state of the art approach of Scully et al. (2010). In their work they used T1w, T2w, and FLAIR sequences in a total of 27 patients, 10 of which were used for training and 17 for testing. Regarding the pre-processing we have included the same steps (co-registration, brain extraction and bias correction) being able to avoid the intensity standardization required in supervised approaches. We have based our approach on three basic characteristics (lesion size, tissue neighborhood of candidate regions, and brain location) while they used 49 morphological features (tissue types and its distance map for each tissue, left-right flipped images to enhance the lesion location, spatial location (x, y, z) to inform about global location, and neighborhoods means and medians). However, only a subset of those features was used at each level of the segmentation by the support vector machines classifier. The comparison at specific points of the ROC curve for both methods showed that our approach obtained similar or even better results. Although we have used a different dataset to evaluate the performance our approach, we have shown an increase in the specificity maintaining similar sensitivity values.

One of the limitations of these automatic WML detection and segmentation approaches are the FN found within the juxtacortical lesions. Even though in our experiments the ratio found was very low, some illustrative examples of this problem are shown in Figure 7. One could address this issue by decreasing the adjustment of the initial threshold (α parameter), but then the FP detections will be higher. This is actually one of the common limitations within the WML segmentations approaches, in which all of them tend to provide a good trade-off between sensitivity and specificity, assuming some FP and FN detections. Notice also that the inter- intra-rater variability could not be assessed in this study since we only had one manual annotation from a single rater.

We believe that the benefits of an unsupervised approach, which allows to avoid the training stage and therefore having manually annotated cases by experts, will help to the community to quantify WML on Lupus patients, specially considering that we provide a public tool which is straightforward to use in SPM8/12.

## 5. Conclusions

In this work we have presented an approach to perform WML segmentation on Lupus patients. We have maintained the same pre-processing pipeline applied in Roura et al. (2015), but now implemented in MATLAB code, fact that facilitates the integration to SPM8/12, the installation and execution. The lesion segmentation process has been modified specially on the application of the refinement constraints due to the difference on the lesion features. Results shown in this manuscript demonstrate the good performance of the approach. The correlation results in both number of lesions and lesion volume, illustrates the validity of the approach as a tool for clinicians when diagnosing Lupus patients or evaluating the disease evolution in patients treated with different therapies.

## Author contributions

ER is the corresponder author who has contributed to develop this work and also to write the manuscript. NS has contributed to annotate the data and also to review the manuscript. AO has contributed to develop and also to write this work. SG has contributed to develop this work. SV has contributed to develop this work and also to review the manuscript. RC has contributed to the acquisition of the database. NB has contributed to the acquisition of the database and also to review the paper. XL has contributed to develop and also to write this work.

### Conflict of interest statement

The authors declare that the research was conducted in the absence of any commercial or financial relationships that could be construed as a potential conflict of interest.

## Footnotes

^1^http://www.nitrc.org/projects/cmtk/

^2^http://eia.udg.edu/salem/slsToolbox/lupus.html

^3^http://www.mathworks.com/matlabcentral/fileexchange/14995-anisotropic-diffusion--perona---malik-

^4^http://www.pmod.com/files/download/v35/doc/pneuro/5674.htm

^5^https://www.nitrc.org/projects/cmtk/
